# The human skin microbiome remains unchanged after 24 h of sunscreen application

**DOI:** 10.1128/aem.01476-25

**Published:** 2025-12-17

**Authors:** Matthew L. Smith, Tim V. Rillaer, Thomas Willmott, Sarah Lebeer, Aline Souza, Catherine A. O’Neill, Andrew J. McBain

**Affiliations:** 1 Division of Musculoskeletal and Dermatological Sciences, School of Biological Sciences, Faculty of Biology Medicine and Health, The University of Manchester 12203, Manchester, United Kingdom; 2 Department of Bioscience Engineering, Laboratory of Applied Microbiology and Biotechnology, University of Antwerp 692679, Antwerp, Belgium; 3 Division of Pharmacy and Optometry, School of Health Sciences, Faculty of Biology, Medicine and Health, The University of Manchester 12203, Manchester, United Kingdom; 4 Croda Europe Ltd., Widnes, Cheshire, United Kingdom; Centers for Disease Control and Prevention, Atlanta, Georgia, USA

**Keywords:** skin microbiome, sunscreen, cosmetic, amplicon sequencing, skincare

## Abstract

To ensure safe, long-term use, topical products should be investigated to understand how they interact with the resident skin microbiota to mitigate potential risk. Sunscreens are essential for protecting skin from UV damage, but their effects on skin-resident microbes have not been well characterized. We examined the impact of two sunscreen formulations (containing titanium dioxide or zinc oxide) on both cultured skin bacteria and the skin microbiomes of human volunteers. No loss of viability was observed after a 2 h exposure to either sunscreen in cultures of *Staphylococcus epidermidis, Staphylococcus capitis, Staphylococcus hominis, Micrococcus luteus,* and *Corynebacterium tuberculostearicum*. The effects of the sunscreens were then studied across the skin microbiomes of 20 human participants. Skin swabs were collected before application and at 1, 6, and 24 h afterward. DNA was extracted and sequenced at the 16S rRNA V4 region, and sequences were denoised and taxonomically assigned using the nf-core/ampliseq pipeline. Across all time points, alpha diversity (Shannon index, Friedman test) and beta diversity (permutational multivariate analysis of variance) remained stable, with no significant differences in beta dispersion. Differential abundance analysis revealed minor fluctuations in some low-abundance genera, identified as likely transient due to their low prevalence, but overall resident community composition was not significantly altered. These findings suggest that short-term sunscreen application does not disrupt the skin microbiome, supporting their safe use from a microbial standpoint. Outcomes from both *in vitro* and *in vivo* experimentation point to the compositional resilience of the skin microbiota to sunscreens.

**IMPORTANCE:**

Understanding how sunscreens affect the skin microbiome is important, given their widespread use and the role of the microbiome in skin health. This study demonstrates that common sunscreens do not significantly alter skin microbiome diversity or viability, including that of the core skin microbiome genera, *Staphylococcus*, *Micrococcus*, *Kocuria*, *Cutibacterium*, and *Corynebacterium*. This highlights the resilience of the skin microbiota and supports the microbiome-safe profile of these products.

## INTRODUCTION

The skin is inhabited by a complex and dynamic community of microorganisms, collectively termed the skin microbiome (1, 2). With modern advancements in microbiome analysis, the skin microbiome has been well-characterized and is known to vary in composition in a site-specific manner across skin (1). Anatomical location is deterministic in selecting for the skin microbiota by creating conditions that are broadly defined as sebaceous, moist, or dry (3). Consequently, differences are often reported in the relative abundance of organisms, contributing to notably different community structures between regions (3–5). Dry skin sites (e.g., forearm) have a characteristically high diversity that includes many species belonging to the genera *Staphylococcus, Micrococcus, Kocuria, Cutibacterium,* and *Corynebacterium* (1, 6). Moist skin sites (e.g., the axilla), with increased localized sweat production, generally have a lower diversity than dry sites and often select for *Corynebacterium* genera. Sebaceous sites are the most selective and therefore have the lowest diversity (1, 7). With increased sebum production at these regions, conditions become increasingly anaerobic and antimicrobial in nature, thus decreasing microbial diversity (1, 7). Many reports indicate *Cutibacterium* genera to be prevalent in sebaceous sites, with research interest often focusing on the role of *Cutibacterium acnes* across the glabella in acne vulgaris (7–10).

This ongoing characterization of the human skin microbiome has resulted in an increased understanding of its role in maintaining healthy skin. The microbiome of the skin protects from pathogenesis, functioning as a direct barrier against infection on the skin’s outermost layer, the stratum corneum (11). It is also reported to maintain skin barrier function, aid in immunological defense, improve wound healing, modulate skin aging, and protect skin from the modulatory effects of ultraviolet radiation (UVR) during sun exposure (12–16). These effects are often correlated with microbiomes that are diverse and stable. Imbalances in microbial diversity, following an overpopulation of select genera, are commonly linked to dermatological conditions such as atopic dermatitis, acne, and psoriasis (17–19).

Probably the biggest extraneous influence on skin is UVR from ambient sunlight. Sunlight consists of ultraviolet A (UVA), UVB, and UVC. Although UVC does not penetrate beyond the ozone layer, both UVA and UVB can interact with skin, possibly resulting in detrimental effects by indirect and direct damage to DNA, proteins, and lipids (20, 21). Excessive sun exposure contributes to erythema (sunburn), basal cell carcinoma, squamous cell carcinoma, melanoma, and skin aging (22). These examples demonstrate the need for effective sun protection strategies, of which sunscreens are an effective tool.

Little is known about how the skin microbiome responds to the ultraviolet filters used in sunscreen. The recommended sunscreen dose includes a large application volume (2 mg/cm^2^) alongside frequent reapplications every 2 h during sun exposure (23, 24). Considering this high dose of sunscreen recommended to consumers, its role in contributing to imbalances in microbial community composition is worthy of study (25).

Therefore, the aim of this research was to investigate the impact of two sunscreens, which contain mineral ultraviolet filters, on the composition of the skin microbiome of 20 healthy human volunteers over a 24 h study period.

## RESULTS

**Fig 1:**
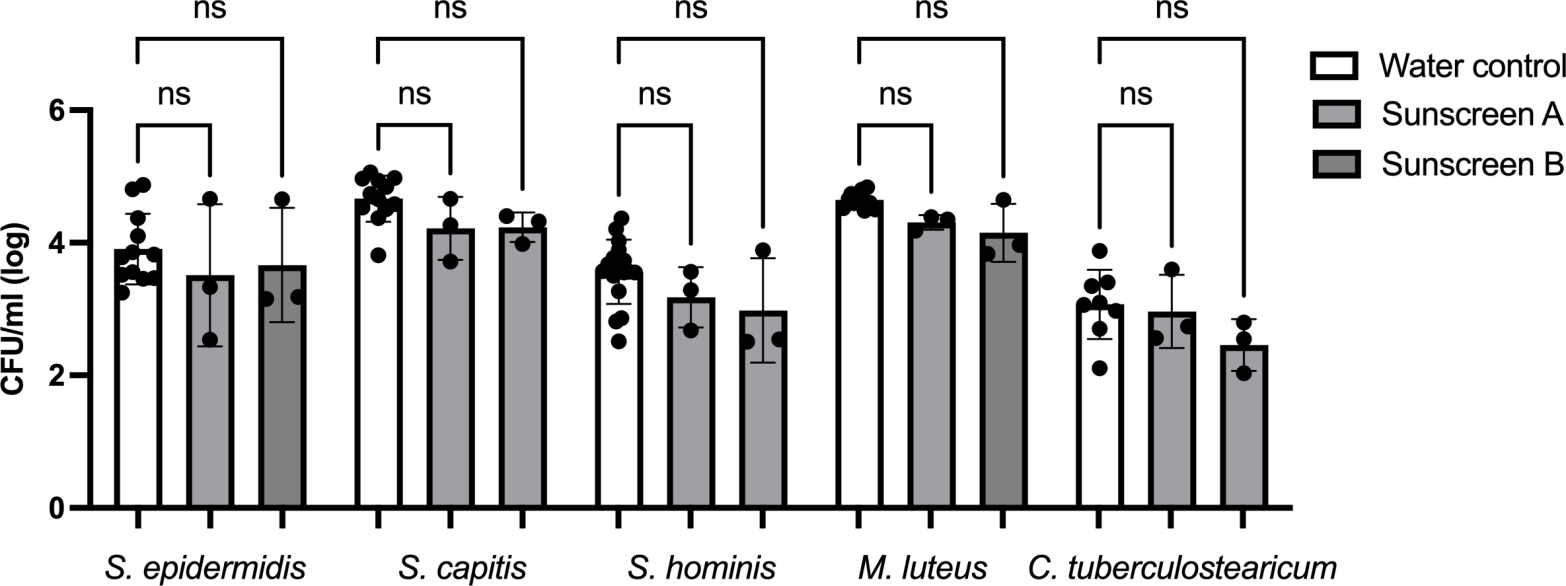
Bacterial viability in response to the application of UVR filters A and B for 2 h. Five single species were individually exposed to either water control, sunscreen A, or sunscreen B for 2 h. Viability was assessed using culture techniques, with tryptone soy agar as a growth medium. The data are displayed as mean CFU/mL (log), with range bars denoting standard deviation. Statistical significance was assessed using analysis of variance, followed by a Tukey *post hoc* test. No statistically significant differences were observed when comparing either sunscreen against its relevant water control for any of the species under test (*, *P* ≤ 0.01) (*n* ≥ 3); ns, not significant.

The direct effects of sunscreen A (titanium dioxide) and sunscreen B (zinc oxide) on the viability of single representative species from the skin microbiome were first assessed using culture-based techniques. Application of sunscreen to *Staphylococcus epidermidis, Staphylococcus capitis, Staphylococcus hominis, Micrococcus luteus,* or *Corynebacterium tuberculostearicum* resulted in no significant difference in the number of viable cells after 2 h of exposure, compared to treatment with a water control (Fig. 1). This indicates these formulations to be microbiologically inert under these conditions.

**Fig 2:**
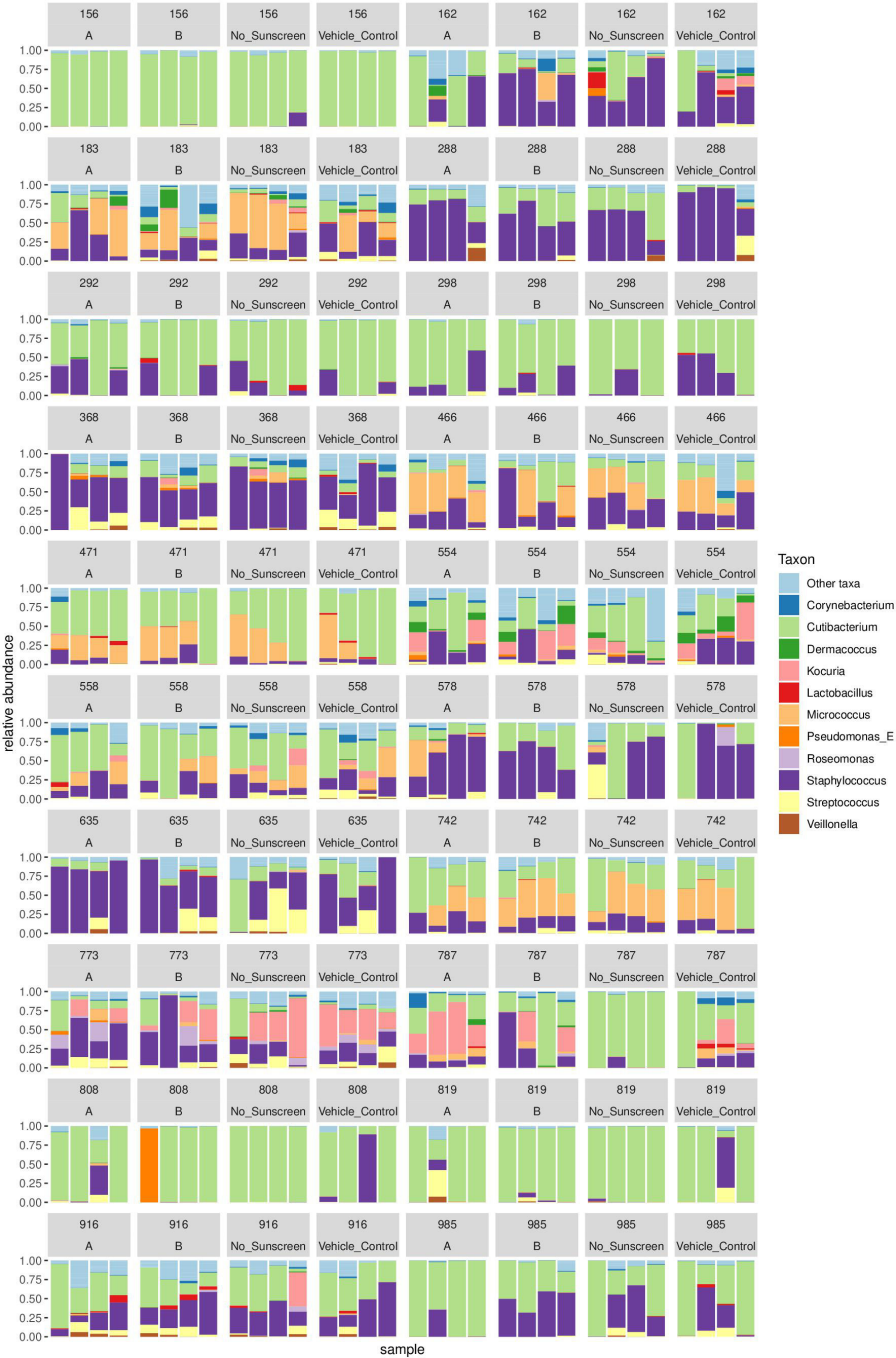
Relative abundance of skin microbiome genera detected on human skin before and 24 h after sunscreen application. Skin swabs were collected from the forearms of 20 participants from regions that had a single 2 mg/cm^2^ application of sunscreen A, sunscreen B, a vehicle control, or were left untreated. Participants were assigned randomly generated numbers to maintain anonymity; these are shown at the top of each graph. Each participant’s data are arranged into four separate plots, grouped linearly. Swabs were collected over the course of four time points. These are arranged as columns under each individual plot, in order from left to right, to display 0 h (before any sunscreen application), 1 h, 6 h, and 24 h after the application of sunscreen. The 11 most frequent genera are color-coded, and their relative abundances are displayed under each condition (*n* ≥ 20).

Analysis of the amplicon sequencing data revealed a large diversity of genera across all skin swab samples, the most common of which includes *Staphylococcus, Micrococcus, Kocuria, Corynebacterium,* and *Cutibacterium* (Fig. 2). Lower-abundance genera, including *Dermacoccus, Roseomonas, Lactobacillus, Pseudomonas, Streptococcus,* and *Veillonella,* were also detected but appear more sporadically among participants. Although the relative abundance of the detected genera varied between individuals, their overall composition remained consistent across treatment conditions.

**Fig 3:**
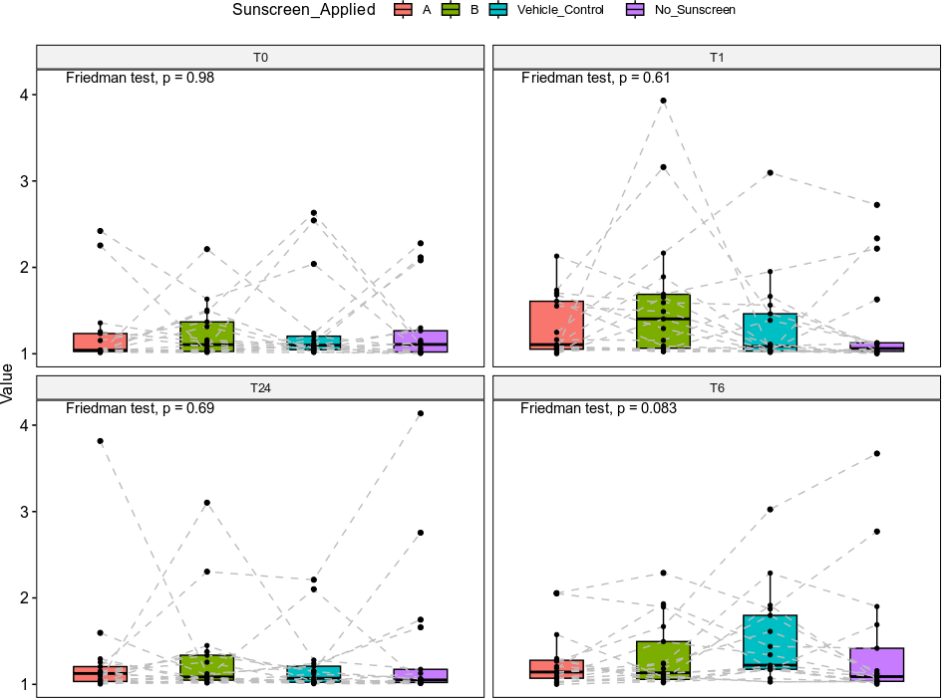
Alpha diversity plots comparing sunscreen-applied skin sites at each time point. Alpha diversity was assessed using the Shannon index distance method and statistically analyzed using a Friedman test. Different skin sites, treated with sunscreen A (red), sunscreen B (green), a vehicle control (blue), or no sunscreen (purple), were plotted onto four individual box plots for each time point, to represent 0 h (T0), 1 h (T1), 6 h (T6), and 24 h (T24) after sunscreen application. Alpha diversity scores (values) for individual participants were plotted individually, and the dashed lines connected points from individual participants, to represent repeated measures. No significant differences in alpha diversity were found between sunscreen conditions, at any of the time points (*, *P* ≤ 0.01).

Alpha diversity was quantified using the Shannon index and statistically analyzed using a Friedman test, comparing the sunscreen A, sunscreen B, the vehicle control, and the “no sunscreen” regions of skin at each time point within participants. No statistically significant differences were found in alpha diversity between each region at 0 h (*P* = 0.60), 1 h (*P* = 0.43), 6 h (*P* = 0.37), or 24 h (*P* = 0.87) after sunscreen application (Fig. 3). This indicates that sunscreen application did not measurably alter within-sample microbial diversity.

**Fig 4:**
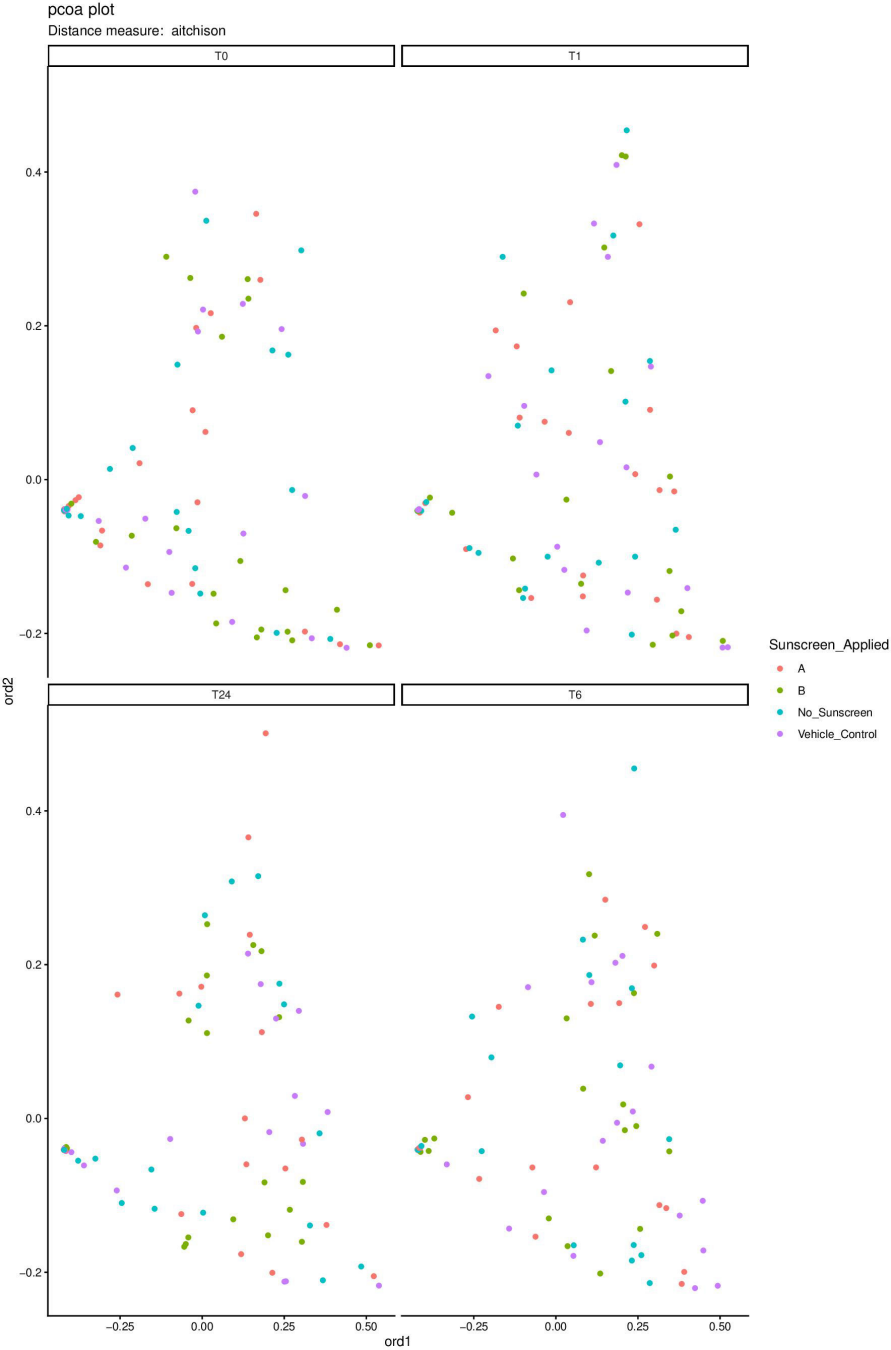
Beta diversity PCoA plot comparing sunscreen-applied skin sites at each time point. Beta diversity was assessed by calculating the Aïtchison dissimilarity. The pairwise relationships were ordinated using PCoA, which shows the distribution of interspecific sample diversity between swabs collected from regions that were applied with either sunscreen A, sunscreen B, a vehicle control, or no sunscreen application. Four PCoA plots were generated, one for each time point, to represent 0 h (T0), 1 h (T1), 6 h (T6), and 24 h (T24) after sunscreen application. The application of sunscreen appears to have no effect on the distribution of sample diversity at any of the four time points.

**Fig 5:**
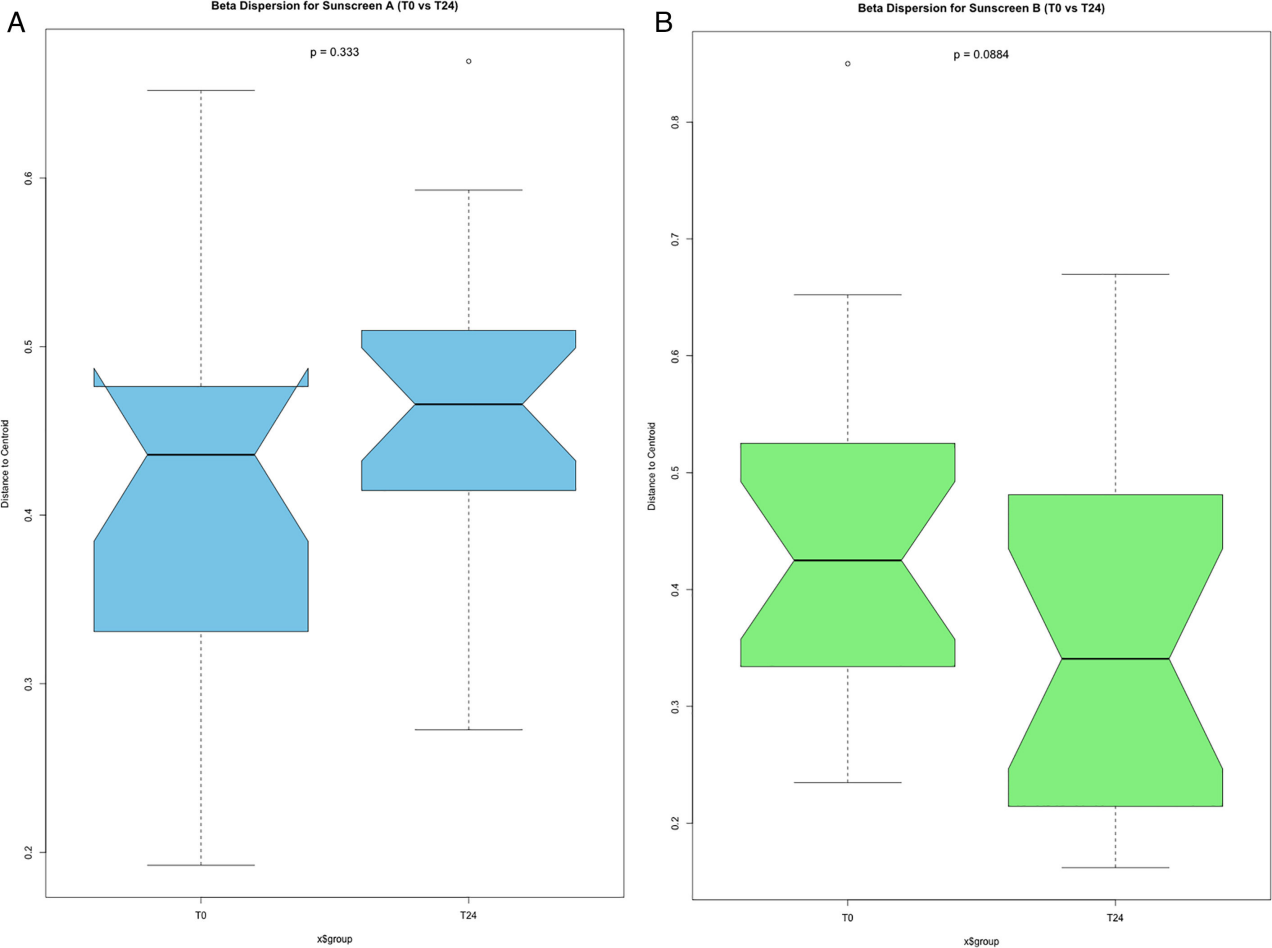
(**A and B**) Beta dispersion box plots comparing skin sites applied with sunscreen A (blue) and sunscreen B (green) at time points T0 and T24. A betadisper test was used to assess sample variability, represented by the distance-to-centroid value. Beta dispersion is compared between samples collected before sunscreen application (T0) and 24 h after sunscreen application (T24), plotted on two different violin plots, for sunscreen A (blue) and sunscreen B (green). Statistical significance was assessed using a permutation-based ANOVA, followed by a Tukey *post hoc* test. No statistically significant difference was found between the beta dispersion values of samples collected either before or 24 h after the application of sunscreen A or B (*, *P* ≤ 0.01).

Beta diversity was assessed using a permutational multivariate analysis of variance (PERMANOVA). A statistically significant difference was found when comparing samples taken from different individuals (*F* = 3.492, *P* = 0.001), indicating high interpersonal variability in skin microbiome composition. In contrast, when grouping samples across all participants and comparing between either the time of collection (*F* = 1.209, *P* = 0.061) or by sunscreen application (*F* = 1.031, *P* = 0.371), no change in beta diversity was observed. Beta diversity was further assessed using the Aïtchison dissimilarity matrix, and the pairwise relations were ordinated using principal coordinate analysis (PCoA) (Fig. 4). This visualization confirmed no clustering for any of the treatment conditions at any of the time points. A beta dispersion analysis was conducted to compare beta diversity variability between the 0 h and 24 h time points. This identified no statistically significant change in beta variability for sunscreen A (*P* = 0.333) or sunscreen B (*P* = 0.088) over this period of 24 h (Fig. 5A and B). Overall, these analyses indicate that sunscreen application did not measurably alter between-sample microbial diversity.

**Fig 6:**
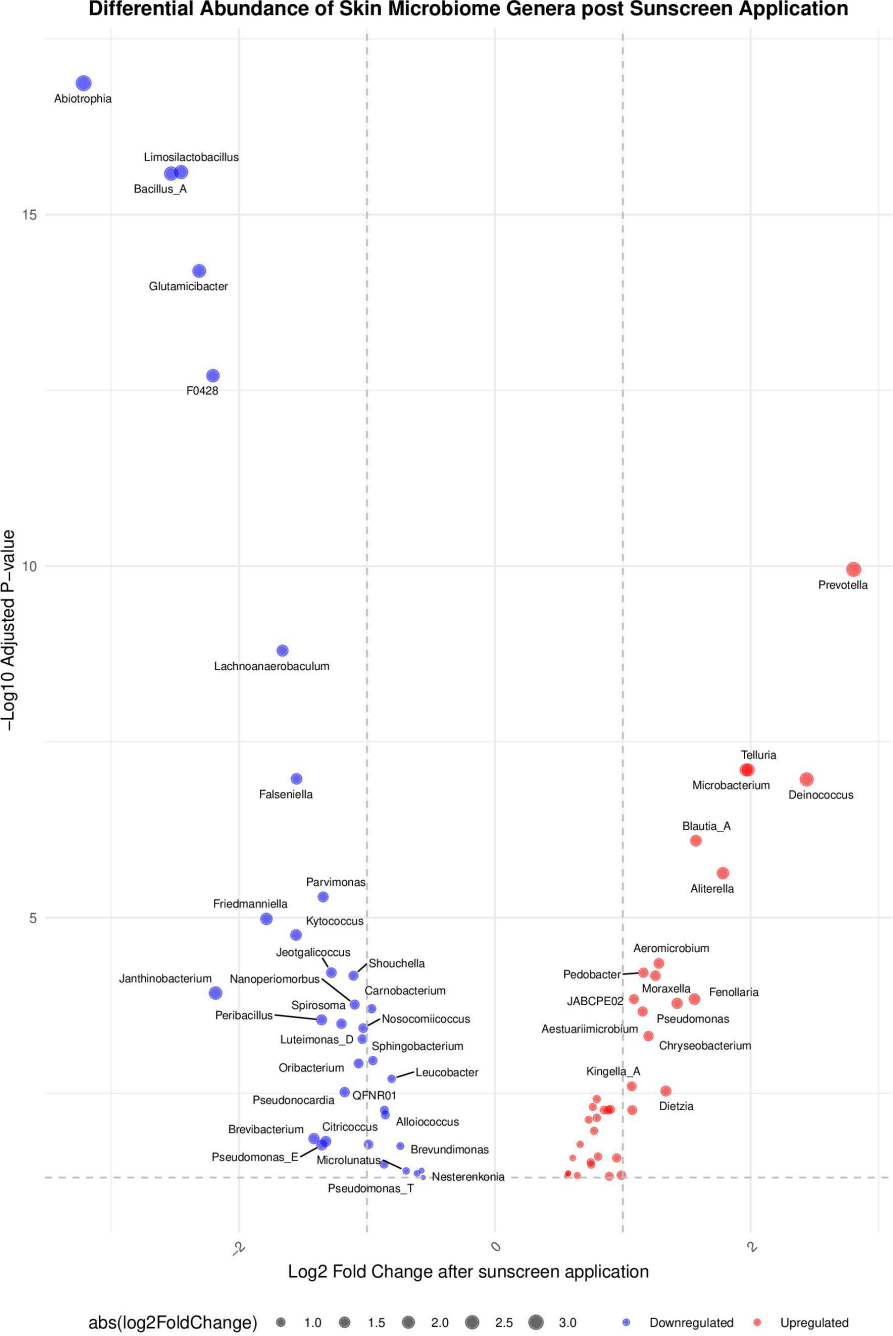
A differential abundance analysis 24 h following a single application of sunscreen. A volcano plot created using the DESeq2 package, which identifies bacterial genera whose abundance changed significantly following sunscreen application. Genera with increased abundances are indicated in red, and those with decreased abundances are indicated in blue (*, *P* ≤ 0.01).

A differential abundance analysis identified a small number of taxa whose relative abundance differed significantly between sunscreen-treated and untreated groups (Fig. 6; Table 1). However, these shifts were limited to low-abundance genera and did not include the 11 most abundant skin-associated taxa. Most of the significantly altered genera were detected in fewer than 10% of all skin swab samples (Fig. S1). Among the 50 genera showing significant changes, only 8 exceeded this 10% prevalence threshold, and none were present in more than 38% of samples. This suggests the low-abundance genera whose relative abundance changed following sunscreen application likely represent transient members of the skin microbiome rather than core residents.

**TABLE 1:** Differential abundance analysis results**^*a*^**

Log_2_ fold change	*P*-value	Adjusted *P*-value	Genus
1.16	4.29e − 06	6.07e − 05	*Pedobacter*
−1.04	6.85e − 05	5.36E − 04	*Luteimonas_D*
−0.95	1.43E − 04	1.08E − 03	*Sphingobacterium*
1.25	5.46e − 06	6.71e − 05	*Moraxella*
−1.11	5.37e − 06	6.71e − 05	*Shouchella*
1.07	9.27E − 04	5.48E − 03	*Arachnia*
−2.53	3.03e − 18	2.60e − 16	*Bacillus_A*
−1.17	4.59E − 04	3.04E − 03	*Pseudonocardia*
−1.66	4.32e − 11	1.59e − 09	*Lachnoanaerobaculum*
−2.45	1.92e − 18	2.48e − 16	*Limosilactobacillus*
−1.28	4.47e − 06	6.07e − 05	*Jeotgalicoccus*
1.09	1.32e − 05	1.45E − 04	*JABCPE02*
0.85	9.35E − 04	5.48E − 03	*Leptotrichia_A*
0.89	9.72E − 04	5.48E − 03	*Novosphingobium*
0.78	2.10E − 03	1.08E − 02	*Agathobacter*
0.80	5.92E − 04	3.82E − 03	*Lentilactobacillus*
0.77	7.85E − 04	4.94E − 03	*Lautropia*
1.16	2.35e − 05	2.17E − 04	*Aestuariimicrobium*
−1.10	1.74e − 05	1.73E − 04	*Nanoperiomorbus*
−1.20	3.76e − 05	3.24E − 04	*Spirosoma*
1.28	2.95e − 06	4.48e − 05	*Aeromicrobium*
0.80	1.31E − 03	7.04E − 03	*Photobacterium*
1.34	4.32E − 04	2.94E − 03	*Dietzia*
−2.18	1.01e − 05	1.19E − 04	*Janthinobacterium*
2.44	4.61e − 09	1.08e − 07	*Deinococcus*
−0.86	9.78E − 04	5.48E − 03	*QFNR01*
−1.03	4.49e − 05	3.74E − 04	*Nosocomiicoccus*
−0.96	2.07e − 05	1.98E − 04	*Carnobacterium*
−1.34	2.75e − 07	5.08e − 06	*Parvimonas*
0.73	1.43E − 03	7.52E − 03	*Fimbriiglobus*
−0.81	2.75E − 04	1.97E − 03	*Leucobacter*
−2.31	9.81e − 17	6.33e − 15	*Glutamicibacter*
−1.79	6.07e − 07	1.04e − 05	*Friedmanniella*
−1.55	1.09e − 06	1.76e − 05	*Kytococcus*
−3.22	5.23e − 20	1.34e − 17	*Abiotrophia*
1.98	2.77e − 09	7.94e − 08	*Telluria*
2.81	2.59e − 12	1.11e − 10	*Prevotella*
1.57	3.74e − 08	8.05e − 07	*Blautia_A*
−1.55	4.11e − 09	1.06e − 07	*Falseniella*
−0.86	1.17E − 03	6.44E − 03	*Alloiococcus*
−2.20	3.79e − 15	1.95e − 13	*F0428*
−1.07	1.61E − 04	1.19E − 03	*Oribacterium*
1.56	1.34e − 05	1.45E − 04	*Fenollaria*
1.96	2.66e − 09	7.94e − 08	*Microbacterium*
1.07	3.61E − 04	2.51E − 03	*Kingella_A*
1.42	1.61e − 05	1.66E − 04	*Pseudomonas*
1.78	1.17e − 07	2.33e − 06	*Aliterella*
1.20	6.01e − 05	4.85E − 04	*Chryseobacterium*
0.90	8.73E − 04	5.36E − 03	*Pseudomonas_O*
−1.35	3.20e − 05	2.85E − 04	*Peribacillus*

^*a*^ A differential abundance analysis was performed using the DESeq2 RStudio package. The bacterial genera whose abundance changed significantly after the application of sunscreen are displayed; genera that showed no statistically significant change in their abundance have not been included in this table. Positive log_2_ fold change values represent an increased abundance following sunscreen application, while negative values represent a decreased abundance (*P *≤ 0.01).

Overall, these findings indicate that the composition and diversity of the resident skin microbiome remained stable following mineral sunscreen application, with significant changes being limited to minor, low-abundance taxa.

## DISCUSSION

This study sought to understand the effects of two mineral/inorganic sunscreens (titanium dioxide and zinc oxide) on the skin microbiome. This is first evidenced with the treatment of live cultures to test the impact of both sunscreens on the viability of five individual and representative species of bacteria. This demonstrated that neither sunscreen impacted microbial viability *in vitro*. This is then further elaborated on by the characterization of the bacterial microbiomes present at each experimental condition, which found that neither titanium dioxide nor zinc oxide has an observable impact on the most abundant members of the skin microbiome. The data generated by both approaches suggest that mineral sunscreens are microbiologically inert, at least over the time scale of this study.

Both titanium dioxide and zinc oxide are commercially available and are common sunscreen ingredients due to their known capacity to protect from sun damage (26). Despite this, little has been done to assess how these common suncare ingredients may interact with the microbiome of the skin. Some variables, such as exposure to sunlight, could not be controlled in this study. To reduce the cumulative impact of these variables over time, the study period was kept short, with samples being collected over 24 h. Although suggesting resilience of the microbiota toward mineral sunscreens in the short term, future study designs should employ frequent applications over longer periods to understand possible long-term effects.

Statistically significant differences were observed when comparing skin microbiome compositions between participants. High interindividual variations are often observed in microbiome studies and are an outcome of the innumerable genetic and environmental variables that select for the microbial communities found across the skin (10, 27). The differences observed between participants are consistent with other skin microbiome studies and are accounted for by a study design, which uses an untreated region of each participant’s skin as their own baseline for comparison (10, 27).

Microbial community composition was assessed before and up to 24 h after sunscreen application using 16S rRNA sequencing. While some statistically significant changes were identified, these occured in a small cohort of less abundant genera, likely transient microbiota, and represent only a small fraction of the known skin microbiome. The transient nature of the impacted group is supported by their low prevalence, with most detected in fewer than 10% of skin swab samples, and none exceeding 38%, a measure used previously to differentiate between transient and resident taxa (28). This interpretation is further supported by many of the identified genera, such as *Prevotella* and *Abiotrophia,* having associations with other body sites, such as the oral and gut microbiomes, and less of a proven association with the skin (29, 30). This distinction could benefit from future longitudinal analyses to better characterize the resident skin microbiome, to more confidently differentiate between low-abundance resident genera and those transiently acquired from the environment.

Previous studies have reported links between the use of cosmetics and observable changes in the skin microbiome (31–33). The two sunscreen formulations used in this study are an exception to these findings, because they caused no significant change in the abundance of most skin microbiome genera. This study has tested anhydrous sunscreens that have been formulated without preservatives to more specifically observe the impacts of the mineral ultraviolet filters themselves. As many commercial sunscreens are formulated with preservatives, they may have impacts on the skin microbiome that are otherwise unaccounted for by this study. Antimicrobial effects have been previously reported in cosmetic preservatives *in vitro* (32). Furthermore, different combinations of preservatives have been shown to exhibit their own unique effects. One example combination (hydroxyacetophenone, phenylpropanol, propanediol, caprylyl glycol, tocopherol, tetrasodium glutamate diacetate) was found to inhibit *Cutibacterium acnes* and *Staphylococcus aureus* without having an inhibitory effect on *Staphylococcus epidermidis* (33). However, another study, utilizing an *in vivo* design, found no significant changes in alpha or beta diversity in skin microbiome samples from participants who applied preservative-containing formulations (34). These examples demonstrate contradictions in the literature surrounding the effects of preservative-containing cosmetics on the human skin microbiota. More research should therefore be conducted to assess whether the results of this study extend to commercial sunscreens that have been formulated with preservatives.

Both UVA and UVB components of UVR have been reported to alter the composition of the skin microbiome (35). To fully understand the relevance of sunscreen application to the skin microbiome, the modulatory effects of UVR and how sunscreens protect from them should be considered. Studies have reported UVR exposure to cause significant changes in beta diversity, demonstrating its ability to alter community composition (36). Specific genera responses have also been reported, finding *Cutibacterium* and *Lactobacilli* to increase and decrease in abundance following UVR exposure, respectively (37, 38). A limited number of studies suggest that sunscreens negate the effects of UVR on the microbiome (37, 38). Sunscreens may therefore have a role in protecting the skin microbiota from the otherwise modulatory effects of UVR. Relevant research into the impact of sunscreens on the skin microbiome has largely focused on these protective effects. This study aimed to provide additional insight by focusing on the impact of sunscreen formulations themselves on the composition of skin microbiome communities.

This study employed an amplicon sequencing approach to characterize the skin microbiome before and after an application of titanium dioxide and zinc oxide sunscreen formulations. Sunscreen applications produced no measurable effect on the resident microbiome, with only minor fluctuations observed, in low-abundance and likely transient genera. Combined with findings that sunscreens have no effect on bacterial viability *in vitro*, these results demonstrate both mineral sunscreens maintain microbial homeostasis at the skin’s surface.

## MATERIALS AND METHODS

### Sunscreen products

Two sun protection factor (SPF) 50 sunscreens were formulated for this protocol, with this SPF chosen to reflect downstream consumer use. A titanium dioxide sunscreen (A) and a zinc oxide sunscreen (B) were each prepared at a 25% (wt/wt) metal oxide concentration to achieve an SPF of 50. Each filter was added to a base containing a mixture of natural waxes (oryza sativa bran wax, sorbitol/sebacic acid copolymer behenate, tribehenin), carrier oil, and dispersing agent, and the formulation was heated to 90°C (Table 2). An additional sunscreen was formulated in the absence of ultraviolet filters, acting as a vehicle control. Each mixture was homogenized using a T25 Ultra-TURRAX (IKA, UK) at 10,000 rpm for 1 minute. The mixture was then allowed to cool to 60°C before being poured into molds and stored at room temperature until future use. Sunscreen ingredients were supplied by Croda International PLC. Liquid emulsion sunscreens were also formulated, in the absence of natural wax, for direct application to bacterial culture.

**TABLE 2:** Sunscreen formulation ingredients**^*a*^**

Ingredient name	Sunscreen A	Sunscreen B	Vehicle control
Isostearyl isostearate	10.70%	13.86%	22.86%
Decyl isostearate (and) isostearyl isostearate	10.37%	13.43%	22.14%
Caprylic/capric triglyceride	7.36%	9.53%	15.71%
Tribehenin	6.69%	8.66%	14.29%
Sorbitol/sebacic acid copolymer behenate	6.69%	8.66%	14.29%
Oryza sativa rice bran wax	3.34%	4.33%	7.14%
Sorbitan oleate (and) polyglyceryl-3 polyricinoleate	1.67%	2.17%	3.57%
TiO_2_ (non-nanoparticle)	53.19% (25% wt/wt active TiO_2_)	0%	0%
ZnO (non-nanoparticle)	0%	39.37 (25% wt/wt active ZnO)	0%

^*a*^ A list of ingredients used in the formulation of sunscreen A, sunscreen B, and the vehicle control, represented as percentages of the total formulation.

### Assessment of bacterial viability in response to sunscreen application

The bacterial cultures used in this study are environmental isolates originally obtained from human skin swabs. Swabs were plated, and single colonies were isolated to establish monocultures. These were subsequently identified using 16SrRNA gene sequencing and stored for future use (39). This work was carried out by Faye Aldehalan in 2020, and ethical approval was provided by the UREC (The University of Manchester) under the ethics reference 2019-6208-10419. These include *Staphylococcus epidermidis, Staphylococcus capitis, Staphylococcus hominis, Micrococcus luteus,* and *Corynebacterium tuberculostearicum.*

Stationary phase bacteria from overnight cultures in tryptone soy broth (Fisher Scientific, UK) were centrifuged at 6,260 × *g* for 10 minutes. The pellet was resuspended into Ringer’s solution (Fisher Scientific, UK) at a concentration of 10^8^ CFU/mL, estimated with optical density measurements (OD_600_) using previously established calibration curves. The bacterial suspension was decanted onto sterile stainless-steel coupons at a volume of 50 µL and dried with a fan in a biological safety cabinet until no visible liquid remained. A sunscreen product was then pipetted onto the dried culture at a volume of 100 µL and left in a biological safety cabinet for 2 h (the recommended time for sunscreen reapplications) (40). Sterile Ringer’s solution was additionally placed onto dried cultures at a volume of 100µL and left to dry in parallel. All coupons were prepared in triplicate.

Coupons, including the volume of either sunscreen or buffer solution applied to them, were then placed into individual sterile glass pots containing 10 mL Ringer’s solution and sterile glass beads. Each pot was shaken by hand for 60 seconds before the contents were serially diluted. Then, 1 mL of the dilution was pipetted into a sterile Petri dish, followed by poured molten (45°C) tryptone soy agar (Fisher Scientific, UK) following a standard pour plate technique. Plates were incubated at 37°C for 1–2 days. Colony counts (CFU/mL) were obtained for all five species, including both sunscreen-treated and buffer-treated cultures for each.

### Participant recruitment

Participants were recruited through an advertisement posted by the University of Manchester research volunteer forum. In total, 20 participants were recruited for this study (Table 3). Involvement only took place once written informed consent had been received and the participants had confirmed that they fell within the inclusion and exclusion criteria. Participants were selected if they were not taking antibiotics for at least 6 months prior to testing; had no known skin conditions or infections; had no known allergies to cosmetics; were not taking large doses of probiotics; and were able to provide informed consent. Participants were additionally pseudonymized, with each being assigned a random three-digit number.

**TABLE 3:** Demographic data for participants recruited for the skin swab study^*a*^

Gender		Age (years)		
Male	Female	21–25	26–30	30+
30%	70%	45%	35%	20%

^*a*^ Gender and age demographic data collected from participants recruited for the skin swab study. These data were collected using pseudonymous questionnaires and represented as percentages from the pool of 20 participants.

### Sunscreen treatment and swab collection

The participant’s forearm was divided into four quadrants. Sterile cotton swabs (Camlab, UK) were soaked in sterile Ringer’s solution (Fisher Scientific, UK) for 10 seconds prior to swabbing. An initial skin swab was taken from each quadrant at the start of the study. Swabs were taken by the experimenter, swiping the swab horizontally and vertically across a 3 cm × 3 cm area on the participant’s forearm for 1 minute. The tip of the swab was then cut off and decanted into a sterile Eppendorf containing glass beads and 350 µL Ringer’s solution. Each of the three sunscreens was then applied to its corresponding quadrant at a volume of 2 mg/cm^2^. Each application was performed once in each region by the experimenter, who applied the sunscreen into the designated area using a gloved hand, until evenly dispersed. The fourth quadrant had no sunscreen products applied, acting as an untreated control. Over the 24 h study period, participants were asked to refrain from washing their forearm but otherwise to carry out their normal daily routines. Swabs were then collected from each quadrant 1, 6, and 24 h after the application of the sunscreens.

### DNA extraction

After collection, swabs were immediately processed for DNA extraction. Eppendorf tubes containing the swab tips were vortexed horizontally using a Vortex-Genie 2 (Fisher Scientific, UK) on the maximum setting for 15 minutes. Samples were then processed for DNA extraction using a DNeasy PowerSoil kit (Qiagen, UK), following manufacturer’s guidelines. Extracted DNA was eluted in a volume of 30 µL before being stored at −80°C.

### Amplicon sequencing

Extracted DNA was loaded into wells of a conical, fully skirted 96-well plate at volumes of 15 µL. DNA sequencing was completed at the Center for Genomics Research, University of Liverpool. Samples undertook a two-step, 40-cycle, V4 PCR amplification, with the use of a FAST liquid handling robot (Formulatrix, UAE). Samples were multiplexed and sequenced using NovaSeq 6000 SP (Illumina, USA), which generates an average of 975,000 read pairs per sample with a target read length of 2 × 150 bp (paired-end).

### Nf-core/ampliseq bioinformatic analysis

After being sequenced, forward and reverse reads were provided in FASTQ format and demultiplexed. The raw reads were then pre-processed, denoised, and amplicon sequence variants (ASVs) were inferred using the nf-core/ampliseq pipeline version 2.10.0 (41). The ASV sequences were classified using a custom database consisting of the GTDB R220 SSU archive supplemented with mitochondrial and chloroplast sequences from the SILVA 138.2 (42, 43). Downstream analyses were performed using the tidytacos (version 1.0.5) R package (RStudio version 4.4.1 [2024-06-14]) (44). Quality control was conducted to remove sequences that had read lengths out of range (outside of 270–320 base pairs).

### Statistical analyses

Alpha diversity was calculated using a Shannon index, and a Friedman test (*P* ≤ 0.01) was used to identify significant effects on alpha diversity from the application of the sunscreen products, across each of the four time points within the participants. Beta diversity was calculated using an Aïtchison dissimilarity matrix and a PERMANOVA test was conducted (*P* ≤ 0.01) to identify statistically significant effects on the beta diversity between participants, the time of swab collection, and the type of sunscreen applied (45). Alpha and beta diversity metrics were calculated at the ASV level, using ASVs generated following quality filtering and denoising. A beta dispersion analysis was then conducted to specifically measure the effects of sunscreen A and sunscreen B on the variability of beta diversity between the 0 h and 24 h time points (46). This was followed by a permutation-based ANOVA and a Tukey *post hoc* test (*P* ≤ 0.01) to confirm statistical significance. A differential abundance analysis was then conducted using the DESeq2 package to identify statistically significant changes in the abundance of bacterial genera following sunscreen applications over the study period (*P* ≤ 0.01) (47). The statistical significance of bacterial viability was assessed using an ANOVA, followed by a Tukey *post hoc* test (*P* ≤ 0.01).

## Data Availability

Sequence data have been made openly available in the NCBI Sequence Read Archive (SRA) under the BioProject accession number PRJNA1271780. This includes all BioSample accessions (SAMN48886790 to SAMN48887116).

## SUPPLEMENTAL MATERIAL

Fig. S1 aem.01476-25-s0001.pdf Prevalence of genera with significantly altered abundance following sunscreen application.

ASM does not own the copyrights to Supplemental Material that may be linked to, or accessed through, an article. The authors have granted ASM a non-exclusive, world-wide license to publish the Supplemental Material files. Please contact the corresponding author directly for reuse.
