# Supplementary material for: The human skin microbiome remains unchanged after 24 h of sunscreen application
Source: Appl Environ Microbiol. 2025 Dec 17;92(1):e01476-25. doi: 10.1128/aem.01476-25 (PMC12838188; doi:10.1128/aem.01476-25)
Supplement: Fig. S1 — Prevalence of genera with significantly altered abundance following sunscreen application. [file aem.01476-25-s0001.pdf]

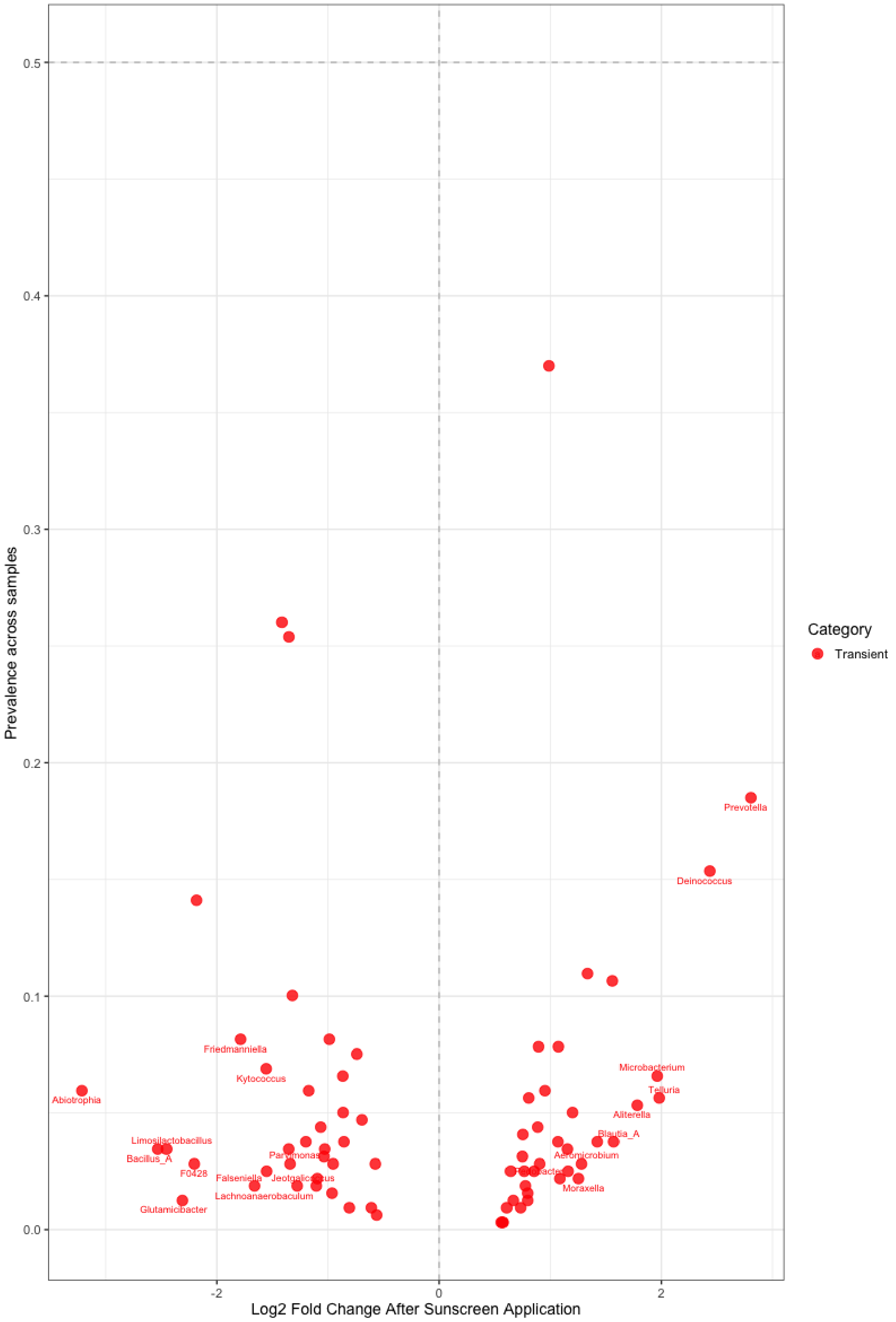

Prevalence across samples
Log2 Fold Change After Sunscreen Application
0.5
0.4
0.3
0.2
0.1
0.0
-2
0
2
Category
Transient
Prevotella
Deinococcus
Friedmanniella
Kytococcus
Abiotrophia
Limosilactobacillus
Bacillus_A
F0428
Falseniella
Lachnoanaerobaculum
Glutamicibacter
Microbacterium
Telluria
Aliterella
Blautia_A
Moraxella

Supplementary Figure 1. Prevalence of genera with significantly altered abundance following sunscreen application. Genera with a significant change in abundance (Log_2 Fold Change) following sunscreen application were plotted against their prevalence (0.1 = 10%) across all skin swab samples. Genera detected in less than <50% of samples were classified as likely transient, while those present in ≥50% were considered likely resident. Positive Log_2 fold change values represent an increased abundance following sunscreen application, while negative values represent a decreased abundance. (*: $p \leq 0.01$).
